# Bayesian network models with decision tree analysis for management of childhood malaria in Malawi

**DOI:** 10.1186/s12911-021-01514-w

**Published:** 2021-05-17

**Authors:** Sanya B. Taneja, Gerald P. Douglas, Gregory F. Cooper, Marian G. Michaels, Marek J. Druzdzel, Shyam Visweswaran

**Affiliations:** 1 Intelligent Systems Program, University of Pittsburgh, 5108 Sennott Square, 210 South Bouquet Street, Pittsburgh, PA 15260 USA; 2 Department of Biomedical Informatics, University of Pittsburgh, Pittsburgh, PA USA; 3 Global Health Informatics Institute, Area 3, Lilongwe, Malawi; 4 Division of Infectious Diseases, Department of Pediatrics, UPMC Children’s Hospital of Pittsburgh, Pittsburgh, PA USA; 5 Faculty of Computer Science, Bialystok University of Technology, Wiejska 45A, 15-351 Bialystok, Poland

**Keywords:** Bayesian network model, Decision tree, Clinical decision support, Childhood malaria, Malawi

## Abstract

**Background:**

Malaria is a major cause of death in children under five years old in low- and middle-income countries such as Malawi. Accurate diagnosis and management of malaria can help reduce the global burden of childhood morbidity and mortality. Trained healthcare workers in rural health centers manage malaria with limited supplies of malarial diagnostic tests and drugs for treatment. A clinical decision support system that integrates predictive models to provide an accurate prediction of malaria based on clinical features could aid healthcare workers in the judicious use of testing and treatment. We developed Bayesian network (BN) models to predict the probability of malaria from clinical features and an illustrative decision tree to model the decision to use or not use a malaria rapid diagnostic test (mRDT).

**Methods:**

We developed two BN models to predict malaria from a dataset of outpatient encounters of children in Malawi. The first BN model was created manually with expert knowledge, and the second model was derived using an automated method. The performance of the BN models was compared to other statistical models on a range of performance metrics at multiple thresholds. We developed a decision tree that integrates predictions with the costs of mRDT and a course of recommended treatment.

**Results:**

The manually created BN model achieved an area under the ROC curve (AUC) equal to 0.60 which was statistically significantly higher than the other models. At the optimal threshold for classification, the manual BN model had sensitivity and specificity of 0.74 and 0.42 respectively, and the automated BN model had sensitivity and specificity of 0.45 and 0.68 respectively. The balanced accuracy values were similar across all the models. Sensitivity analysis of the decision tree showed that for values of probability of malaria below 0.04 and above 0.40, the preferred decision that minimizes expected costs is not to perform mRDT.

**Conclusion:**

In resource-constrained settings, judicious use of mRDT is important. Predictive models in combination with decision analysis can provide personalized guidance on when to use mRDT in the management of childhood malaria. BN models can be efficiently derived from data to support clinical decision making.

**Supplementary Information:**

The online version contains supplementary material available at 10.1186/s12911-021-01514-w.

## Background

Malaria is a mosquito-borne infectious disease that is a major cause of death in children under five years old in low- and middle-income countries (LMICs). Accurate diagnosis and management of malaria can help reduce the burden of childhood morbidity and mortality in LIMCs. In Malawi, the overall prevalence of malaria in children under five is 24%, with the prevalence being as high as 48% in rural areas [1]. Among the several malarial parasites, Plasmodium falciparum causes 98% of all malarial infections and all instances of severe illness and death in Malawi [1]. Management of childhood malaria in Malawi is provided at health posts and health centers that serve as primary healthcare facilities, district hospitals that serve as secondary healthcare facilities, and central hospitals that serve as tertiary centers of care. Management of common childhood illnesses, such as malaria, is provided mainly at the health posts by community-based healthcare workers known as Health Surveillance Assistants (HSAs), and at health centers that are staffed with HSAs and medical assistants. For the majority of the population, health posts and health centers in rural areas serve as the primary sites of care [1].

Historically, in LMICs, presumptive treatment of fever with anti-malarial drugs was common. The current standard for the management of childhood malaria is defined in a set of clinical guidelines developed by the World Health Organization (WHO) [2]. Based on these guidelines, a child presenting with fever and suspected of having malaria should have the diagnosis confirmed from a drop of blood using either microscopic examination or malaria rapid diagnostic test (mRDT) that rapidly detects antigens derived from malarial parasites. The mRDT is a useful and less expensive alternative to microscopy. In 2010, Malawi adopted the WHO guidelines as national policy and instituted the use of mRDT for suspected malaria as standard practice. The WHO-recommended treatment for malaria caused by P. falciparum is artemisinin-based combination therapy (ACT) that combines two active ingredients with different mechanisms of action. Malawi extensively uses ACT for the treatment of childhood malaria. Malawi has made significant efforts to provide community-based care for childhood malaria by adopting mRDT and ACT coupled with their national distribution, and these efforts have led to a decline in the disease burden [3]. However, several challenges remain that hinder the effective management of malaria in rural Malawi.

Health posts in rural Malawi are characterized by the limited availability of resources, unavailability of diagnostic testing facilities, and lack of clinicians [4]. In a study conducted in 2017 in Malawi, Klootwijk et al. [5] reported a lack of microscopy facilities in the rural health centers that were surveyed. mRDTs and HIV tests are typically the only diagnostic tests available at the health posts and rural health centers [6]. Even so, mRDTs and ACT drugs are in limited supply in rural areas, especially during the malaria season. The Malawi Service Provision Assessment (SPA) survey reported that mRDTs are available only in 85% of the facilities. Hospitals, which are located in urban centers, have the highest proportion available (95%), and health posts, which are located in rural areas, the lowest (19%) [6]. Common reasons for stockouts include late and inaccurate reporting of supplies, drug pilferage, and overprescribing of anti-malarial and antibiotic drugs [4, 7]. As HSAs are encouraged to adhere to the WHO guidelines, the unavailability of mRDTs leads to one of three common responses at the health posts. The child may be referred to a secondary health center or a tertiary hospital; the HSA treats the child presumptively with ACT drugs if the child is febrile and the drugs are in stock; or in the worst case, the health post stays closed while mRDTs are out of stock. Often, the guardians of the child cannot arrange transportation to the referred site, and the child is not treated [5]. When available, mRDTs and ACT drugs are provided free of cost to patients at all healthcare facilities in Malawi. Data on the affordability of drugs shows that a single course of treatment is unaffordable for a major part of the population [8]. This can be a problem if the guardians are advised to purchase ACT drugs on the market when the drugs are unavailable at the healthcare facilities. Given the high volume of patients and increasing non-adherence to traditional paper-based management guidelines [9], it is imperative to provide support to the healthcare workers for accurate diagnosis and treatment with sustainable resource use.

Technological advances can help tackle some of the above challenges. The promise of artificial intelligence and statistical models for healthcare in LMICs has recently begun to see the light [10]. While clinical decision support systems that use statistical models are available in high-income countries, the transfer of these technologies to LMICs is impractical due to the unique challenges in resource-constrained countries. The distinct needs, diseases, demographics, and standards of care in LMICs call for a different approach to personalized and affordable medicine by adopting tools specifically designed for use in these areas [11]. Prior attempts to develop clinical decision support in Malawi have focused on implementing electronic versions of existing guidelines rather than personalized evidence-based algorithms [12, 13]. There is a significant lack of diagnostic support for the healthcare workers in these applications.

A recent review of electronic clinical decision algorithms (eCDAs) in LMICs identifies the lack of effective, integrated diagnostic tools as a contributing factor to childhood morbidity and mortality [13]. In addition to better diagnosis of diseases and support for rational use of drugs, the review identifies components of an eCDA that are crucial to close gaps in the primary care management systems in low-resource countries. These include algorithms for specific regions, openly available evidence-based content, automated data collection for monitoring and evaluation, and syndromic-based surveillance systems [13]. One promising type of model that can be used for the diagnosis of diseases using data is the Bayesian network (BN). A BN probabilistically models associations between variables such as a disease and its clinical features [14, 15], and can be used to predict the presence of the disease. BN models have been developed to aid diagnosis and risk assessment in many diseases [16–20], and a wide range of algorithms are available that automatically learn BN models from data [21–23].

Our long-term goal is to implement a clinical decision support system for childhood malaria in Malawi to aid in the management of malaria, especially where mRDT is unavailable or in limited supply. In this study, we derived several BN models to predict childhood malaria from data obtained from Malawi, and we compared them to other commonly used statistical models. Further, we provide an illustrative decision analysis that integrates predictions from our BN models with the costs of available alternatives for management.

## Materials and methods

We first describe the Malawi Service Provision Assessment (SPA) [6] dataset, followed by the methods for the development and evaluation of BN models and the comparison of other statistical models. Finally, we describe the details of the decision tree that we developed for decision analysis.

### The SPA dataset

The SPA survey was conducted between July 2013 and February 2014 by the Ministry of Health of Malawi, with support from the Demographic and Health Surveys (DHS) Program, to assess the status of health facilities and quality of healthcare in Malawi. Data were collected from 1,060 facilities comprised of 97 hospitals, 489 health centers, 55 dispensaries, 369 clinics, and 28 health posts across three major regions in the country, and are representative at the national level by facility type and managing authority [6]. These data have been used previously in studies to assess the quality of care and treatment for pneumonia in Malawi [24] and are freely available from the DHS program [25].

The survey dataset contains observations on 3,441 encounters with children aged 2 to 59 months presenting to an outpatient healthcare facility. For each encounter, the data contains demographic details (age, date of birth, and sex), clinical features (duration of illness, fever, diarrhea, anemia, etc.), mRDT result (if available), and the provider’s diagnosis.

### Data preprocessing

We assumed the result of the mRDT that is recorded in the dataset to be the gold standard malaria diagnosis. The mRDT has high sensitivity and specificity (0.997 and 0.995 respectively) for the diagnosis of malaria [26] and is recommended for confirmation of disease by both the WHO and Malawi’s malaria management guidelines [27]. Thus, if the mRDT result was positive, we considered malaria to be present, and if the test result was negative, we considered malaria to be absent. This variable is referred to as ‘malaria’ or ‘malaria diagnosis’ in the following sections.

While it would have been ideal to have the mRDT result for each encounter in the dataset, this is not the case. Of the 3,441 encounters, an mRDT result was recorded for only 1,139 encounters, and we restricted our analyses to only these encounters. Table 1 shows the variables that we identified to include for modeling. These variables were chosen based on their inclusion in childhood illness management guidelines [2] as well as on expert domain knowledge. Two of the variables are continuous (age and duration of illness), and the remaining variables are categorical. We discretized the continuous variables since the BN algorithms we used are designed for discrete variables. We discretized age by months (< 2, 2–12, 13–24, 25–60, > 60) based on the varying epidemiology of the disease in children of different ages. We discretized the duration of illness by the number of days, as shown in Table 1. Every predictor variable had one or more missing values, and we denoted them with a special value called ‘Unknown’. Thus, we explicitly modeled the absence of data. The target variable, malaria, is binary, taking the values ‘Positive’ or ‘Negative’ that represent the mRDT result.

**Table 1:** Variables and values that were included in the models

Variable	Values
*Target variable*	
Malaria	Present
	Absent
*Predictor variable*	
Age	Less than 2 months
	2–12 months
	13–24 months
	25–60 months
	Over 60 months
	Unknown
Duration of illness	Less than or equal to 2 days
	3–15 days
	16–30 days
	Over 30 days
	Unknown
Conscious	Yes
	No
	Unknown
Anemia	Present, Absent, Unknown
Convulsions	Present, Absent, Unknown
Cough or difficulty breathing (CDB)	Present, Absent, Unknown
Diarrhea	Present, Absent, Unknown
History of fever	Present, Absent, Unknown
Fever (temperature > 37.5 °C)	Present, Absent, Unknown
Lethargy	Present, Absent, Unknown
Malnutrition	Present, Absent, Unknown
Unable to feed	Present, Absent, Unknown
Vomiting	Present, Absent, Unknown

### Bayesian network models

A BN model is a probabilistic graphical model that is specified by a graphical structure and a set of numerical parameters [14]. The graphical structure consists of nodes representing variables and arcs denoting associations between pairs of variables. In this paper, we use nodes and variables interchangeably. Each node in the network has an accompanying conditional probability table that constitutes the parameters of the node. A BN model can be used as a classifier where the model provides the posterior probability distribution of a target node (such as a disease diagnosis) given the values of all other nodes (such as clinical features) in the network [23]. Several approaches are available to construct a BN model. In the first approach, both the structure and the parameters are specified manually using expert knowledge. In the second approach, the structure is specified manually, and the parameters are estimated from data. In a third approach, both the structure and parameters are automatically estimated from data; a variety of algorithms have been developed to automatically derive BN models in this way. In this study, we used the second and third approaches to develop two BN models for the prediction of malaria using the GeNIe Modeler tool [28] from the variables listed in Table 1. For the first model (manual model), we manually specified the structure based on domain knowledge and computed the parameters of each node using the GeNIe Modeler. For the second model, we used the GeNIe Modeler to automatically derive the structure of a Tree Augmented Naïve Bayes model (described later) and the parameters of each node in the model. For both models, we used the GeNIe Modeler to compute the parameters of each node from the dataset by estimating the conditional probability distribution of the node given the values of its parent nodes [23].

#### Manual model

Based on domain knowledge of malaria from experts and the literature, for the manual model, we modeled clinical features as conditionally independent of each other given malaria. Specifically, a clinical feature that was a symptom or a sign was represented as a child of the malaria node to create a Naïve Bayes-like structure. A feature that was not a sign or a symptom was represented as a parent of the malaria node. For example, a sign such as convulsions was represented as a child of malaria with the arc directed from malaria to convulsions. This encodes clinical knowledge that malaria can cause convulsions. As another example, age was represented as a parent of malaria, with the arc directed from age to malaria. This denotes knowledge that younger children may be more vulnerable to contracting malaria than older children. In a Naïve Bayes disease model, each sign or symptom node has a single incoming arc from the disease node with no arcs among them.

#### Tree Augmented Naïve Bayes model

While the manual model is simple and interpretable, the conditional independence assumption may be overly simplistic. Hence, we developed a second model by automatically deriving a Tree Augmented Naïve Bayes (TAN) model using the GeNIe Modeler. The TAN model extends the Naïve Bayes model by allowing arcs among child nodes [21]. For example, in a Naïve Bayes model, diarrhea and convulsions are linked only by incoming arcs from the malaria node, while in the TAN model, an additional arc may be included from diarrhea to vomiting that implies vomiting is associated with both malaria and diarrhea. The TAN algorithm in GeNIe Modeler enables efficient learning of both the structure and the parameters of a TAN model.

### Comparison models

For comparison with the diagnostic predictions of BN models, we derived several commonly used statistical models, including logistic regression and random forest, to predict malaria. Instead of discretizing the continuous variables, age and duration of illness, we scaled them so that the values had unit variance; when a variable had missing values, we imputed its value as the mean of its non-missing values. We treated the categorical variables in the same way as for the BN models. We derived and evaluated the models using the scikit-learn library [29] in Python. The logistic regression model was derived using the L2 penalty, and the value of the regularization hyperparameter was determined using a search over seven possible values (0.001, 0.01, 0.1, 1, 10, 100, 1000). The hyperparameters (and the values over which the search was performed) for the random forest model included the number of trees in the forest (100, 200, 500), a criterion for the split (“gini”, “entropy”), maximum depth of the tree [4–8], and the number of features (square root of total features, log of total features, total features).

### Derivation and evaluation

We derived and evaluated the manual BN, TAN, logistic regression, and random forest models using tenfold cross-validation. The dataset was divided into 10 folds, stratified on malaria diagnosis. Over 10 iterations, each fold was used as a test set in turn, and the remaining folds were combined to form the training set. For the manual BN model, we estimated the parameters of the model using tenfold cross-validation while the structure was fixed across all iterations. For the TAN model, we estimated both the structure and parameters using tenfold cross-validation. For the logistic regression and random forest models, during each iteration of cross-validation, the hyperparameters were chosen using the training set.

During each iteration of cross-validation, we applied the models to predict the probability of malaria in the test set. Using these predictions, we computed the area under the Receiver Operating Characteristic curve (AUC). The AUC value indicates the diagnostic discrimination performance of the model, where perfect performance has an AUC of 1. Then, we converted the probability into a binary prediction of malaria present or absent by using two probability thresholds, including the default threshold of 0.5 and an optimal threshold obtained by maximizing the Youden Index. The threshold that maximizes the Youden Index is the threshold that optimizes the model’s ability when equal weight is given to sensitivity and specificity [30]. With the binary predictions, we computed balanced accuracy (BAC), sensitivity, specificity, and the net reclassification improvement (NRI) at both thresholds. BAC is the average of sensitivity and specificity and is more useful than accuracy when the proportion of the target values are imbalanced. NRI quantifies how well a new model correctly reclassifies children with and without malaria compared to a baseline model [31]. NRI is computed as:

(sensitivity of new model − sensitivity of baseline model) + (specificity of new model − specificity of baseline model).

For statistical comparisons, we used the DeLong’s test to compare AUCs of two models [32], the paired two-sample Wilcoxon test to compare BACs of a pair of models [33], and McNemar’s Chi-Square test to compare sensitivities and specificities of two models [33].

### Decision tree development

To conserve the use of mRDT in a resource-constrained setting like a rural health post in Malawi, we developed a decision tree to compare the consequences of using and not using the mRDT. The decision tree integrates the probability of having malaria (that is obtained from a predictive model) with the costs of testing and treatment and identifies the optimal decision (relative to a set of probabilities and utilities) − to use mRDT or not − in a specific patient.

**Fig. 1:**
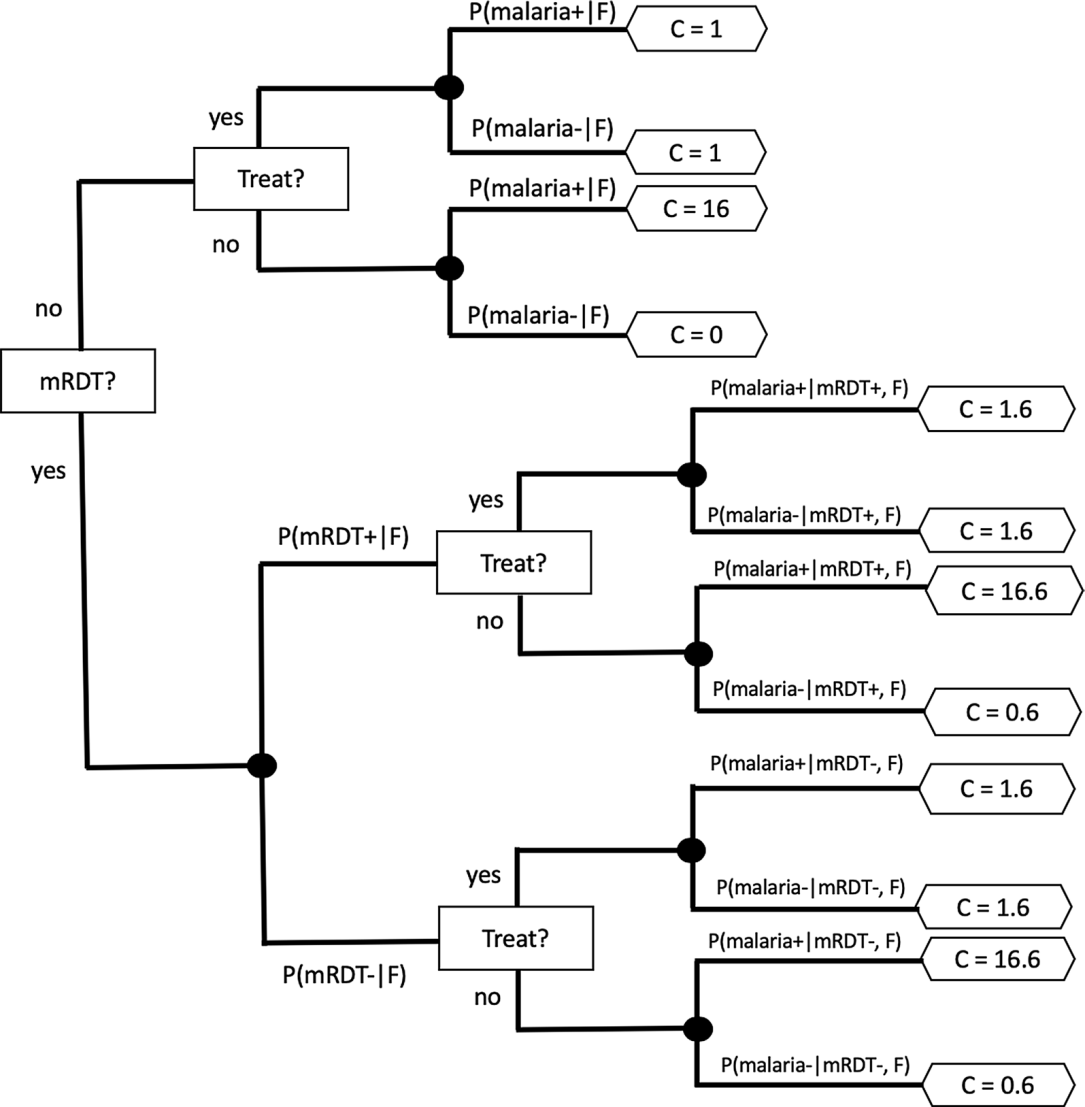
Illustrative decision tree that integrates predictions from a model with example costs. Malaria+ and malaria- represent malaria present and absent respectively, F refers to clinical features of the patient, and C is the associated cost

The decision tree that we developed is shown in Fig. 1 and uses a standard approach to model sequential decisions [34]. The decision is driven by the expected costs of testing and treatment that are denoted by ‘mRDT?’ and ‘Treat?’ nodes. We calculated the expected cost of the [mRDT? = no] branch using the probability of malaria from a predictive model and costs associated with each decision as

$${\text{Expected cost of [mRDT? = no] = min(1}}{\text{.0*P(malaria+|F) + 1}}{\text{.0*P(malaria-|F), 16*P(malaria+|F))}}{.}$$

In Fig. 1, *P*(malaria+|F) is the probability that malaria is present given the clinical features of the patient. *P*(malaria-|F) is the probability that malaria is absent given the features. The costs (shown in the hexagons) in the decision tree are from the perspective of a payer of healthcare costs, such as the government of Malawi, and depend on the resources used, including mRDT and ACT drugs. We used the following costs based on the literature: an mRDT costs US $0.60 [8] and a course of ACT for uncomplicated malaria costs US $1.00 [35]. We estimated the cost of mistakenly not treating a child with malaria at US $16.60 based on the assumption that the cost may go up to 10 times the cost of mRDT and ACT drugs for uncomplicated malaria if the untreated disease becomes severe, resulting in hospital admission.

We computed the expected cost of the [mRDT? = yes] branch as.

$$\begin{aligned} & {\text{Expected cost of [mRDT? = yes] = P(mRDT+|F)*(Expected Cost of [Treat?] when mRDT+)}} \\ & \quad \quad \quad \quad \quad \quad \quad \quad \quad \quad \quad \quad \quad \quad \quad {\text{ + P(mRDT-|F)*(Expected Cost of [Treat?] when mRDT-),}} \\ \end{aligned}$$

where

$$\begin{aligned} & {\text{Expected cost of [Treat?] when mRDT+ = min(1}}{\text{.6*P(malaria+|mRDT+,F) + 1}}{\text{.6*P(malaria-|mRDT+,F), 16}}{\text{.6*P(malaria+|mRDT+,F)}} \\ & \quad \quad \quad \quad \quad \quad \quad \quad \quad \quad \quad \quad \quad \quad \quad \quad \quad {\text{ + 0}}{\text{.6*P(malaria-|mRDT+,F)),}} \\ \end{aligned}$$

and

$$\begin{aligned} & {\text{Expected cost of [Treat?] when mRDT- = min(1}}{\text{.6*P(malaria+|mRDT-,F) + 1}}{\text{.6*P(malaria-|mRDT-,F),16}}{\text{.6*P(malaria+|mRDT-,F)}} \\ & \quad \quad \quad \quad \quad \quad \quad \quad \quad \quad \quad \quad \quad \quad \quad \quad \quad {\text{ + 0}}{\text{.6*P(malaria-|mRDT-,F))}}{\text{.}} \\ \end{aligned}$$

In the above equations, *P*(malaria+|mRDT+, F) is the probability of malaria being present given that the mRDT result is positive and the clinical features of the patient, and *P*(malaria-|mRDT−, F) is the probability of malaria being absent given that the mRDT result is negative and the clinical features of the patient. *P*(mRDT+|F) and *P*(mRDT-|F) represent the probabilities of mRDT being positive or negative, respectively, given the clinical features of the patient. These probabilities are obtained from a model such as the manual BN model and are assumed to be equal to *P*(malaria+|F) and *P*(malaria-|F) respectively. We have assumed that a positive result on the mRDT is equivalent to the child having malaria since the test is so accurate.

We also performed a sensitivity analysis to determine how dependent the strategy selection is on the probability of malaria. We varied the probability of malaria, *P*(malaria+|F) or *P*(mRDT+|F), from 0 to 1 and calculated the expected costs of using and not using the mRDT to determine the probability ranges in which a child may be treated based on clinical features alone without performing mRDT in order to minimize cost.

## Results

In this section, we discuss the characteristics of the SPA dataset, followed by a description of the BN models. We compare the predictive performance of all the models developed. Finally, we present the sensitivity analysis based on the decision tree.

### Characteristics of the dataset

The dataset that we used for modeling contains 1,139 encounters, 13 predictor variables, and the target variable (see Table 2). Malaria was present in 415 (36.4%) of the encounters. The most common age category of the children was from 24 to 60 months (35.7%). The duration of illness varied from 0 days to over 30 days, although the duration period of 0 to 2 days was the most common (51.7%). The most common clinical features were a history of fever (69.3%) and CDB (62.8%) followed by vomiting (29.1%) and diarrhea (26.3%). The percentage of ‘Unknown’ values ranged from 0 to 6.4%, with a history of fever having the highest percentage missing and anemia and malnutrition having the lowest.

**Table 2:** Summary of the dataset

Variable	Number of encounters (%)	Number of encounters (%)	Number of encounters (%)
	Malaria: present (N = 415)	Malaria: absent (N = 724)	Total (N = 1139)
**Predictor variable**			
Age (in months)			
2–12	103 (24.82)	276 (38.12)	379 (33.27)
12–24	117 (28.19)	177 (24.45)	294 (25.81)
24–60	178 (42.89)	229 (31.63)	407 (35.73)
Other	6 (1.45)	15 (2.07)	21 (1.84)
Unknown	11 (2.65)	27 (3.73)	38 (3.34)
Duration of illness (in days)			
≤ 2	214 (52.57)	375 (51.80)	589 (51.71)
3 to 15	186 (44.83)	316 (43.65)	502 (44.07)
15 to 30	5 (1.20)	6 (0.83)	11 (0.97)
Unknown	10 (2.41)	27 (3.73)	37 (3.25)
Conscious			
Yes	397 (95.66)	679 (93.78)	1076 (94.47)
No	4 (0.96)	8 (1.10)	12 (1.05)
Unknown	14 (3.37)	37 (5.11)	51 (4.48)
Anemia			
Present	38 (9.16)	55 (7.60)	93 (8.17)
Absent	377 (90.84)	669 (92.4)	1046 (91.83)
Unknown	0	0	0
Convulsions			
Present	20 (4.82)	35 (4.83)	55 (4.83)
Absent	386 (93.01)	661 (91.30)	1047 (91.92)
Unknown	9 (2.17)	28 (3.87)	37 (3.25)
Cough or difficulty breathing (CDB)			
Present	258 (62.17)	457 (63.12)	715 (62.77)
Absent	143 (34.46)	230 (31.77)	373 (32.75)
Unknown	14 (3.37)	37 (5.11)	51 (4.48)
Diarrhea			
Present	109 (26.27)	190 (26.24)	299 (26.25)
Absent	297 (71.57)	506 (69.89)	803 (70.50)
Unknown	9 (2.17)	28 (3.87)	37 (3.25)
Fever (temperature > 37.5 C)			
Present	148 (35.66)	159 (21.96)	307 (26.95)
Absent	248 (59.76)	523 (72.24)	771 (67.69)
Unknown	19 (4.58)	42 (5.80)	61 (5.36)
History of fever			
Present	313 (75.42)	476 (65.75)	789 (69.27)
Absent	78 (18.80)	199 (27.49)	277 (24.32)
Unknown	24 (5.78)	49 (6.77)	73 (6.41)
Lethargy			
Present	97 (23.37)	131 (18.09)	228 (20.02)
Absent	309 (74.46)	566 (78.18)	875 (76.82)
Unknown	9 (2.17)	27 (3.73)	36 (3.16)
Malnutrition			
Present	6 (1.45)	5 (0.69)	11 (0.97)
Absent	409 (98.55)	719 (99.31)	1128 (99.03)
Unknown	0	0	0
Unable to feed			
Present	59 (14.22)	78 (10.77)	137 (12.03)
Absent	347 (83.61)	619 (85.50)	966 (84.81)
Unknown	9 (2.17)	27 (3.73)	36 (3.16)
Vomiting			
Present	132 (31.81)	199 (27.49)	331 (29.06)
Absent	274 (66.01)	496 (68.52)	770 (67.60)
Unknown	9 (2.17)	29 (4.02)	38 (3.34)

### Description of Bayesian network models

**Fig. 2:**
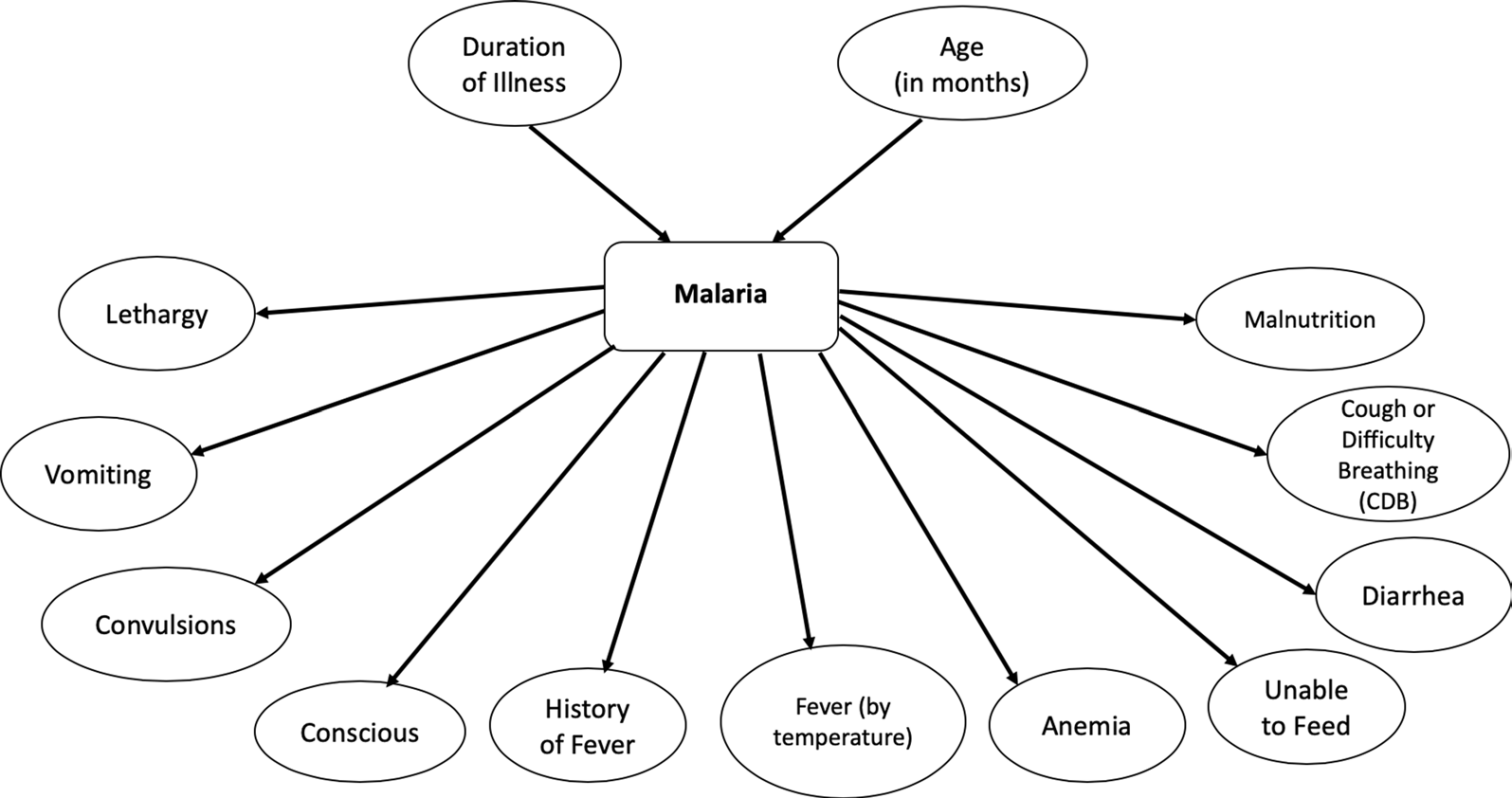
The manual BN model

The manual BN model is shown in Fig. 2. The model contains 14 nodes with 13 arcs. The variables duration of illness and age are modeled as parents of the malaria node, while the clinical features are modeled as children of the malaria node.

**Fig. 3:**
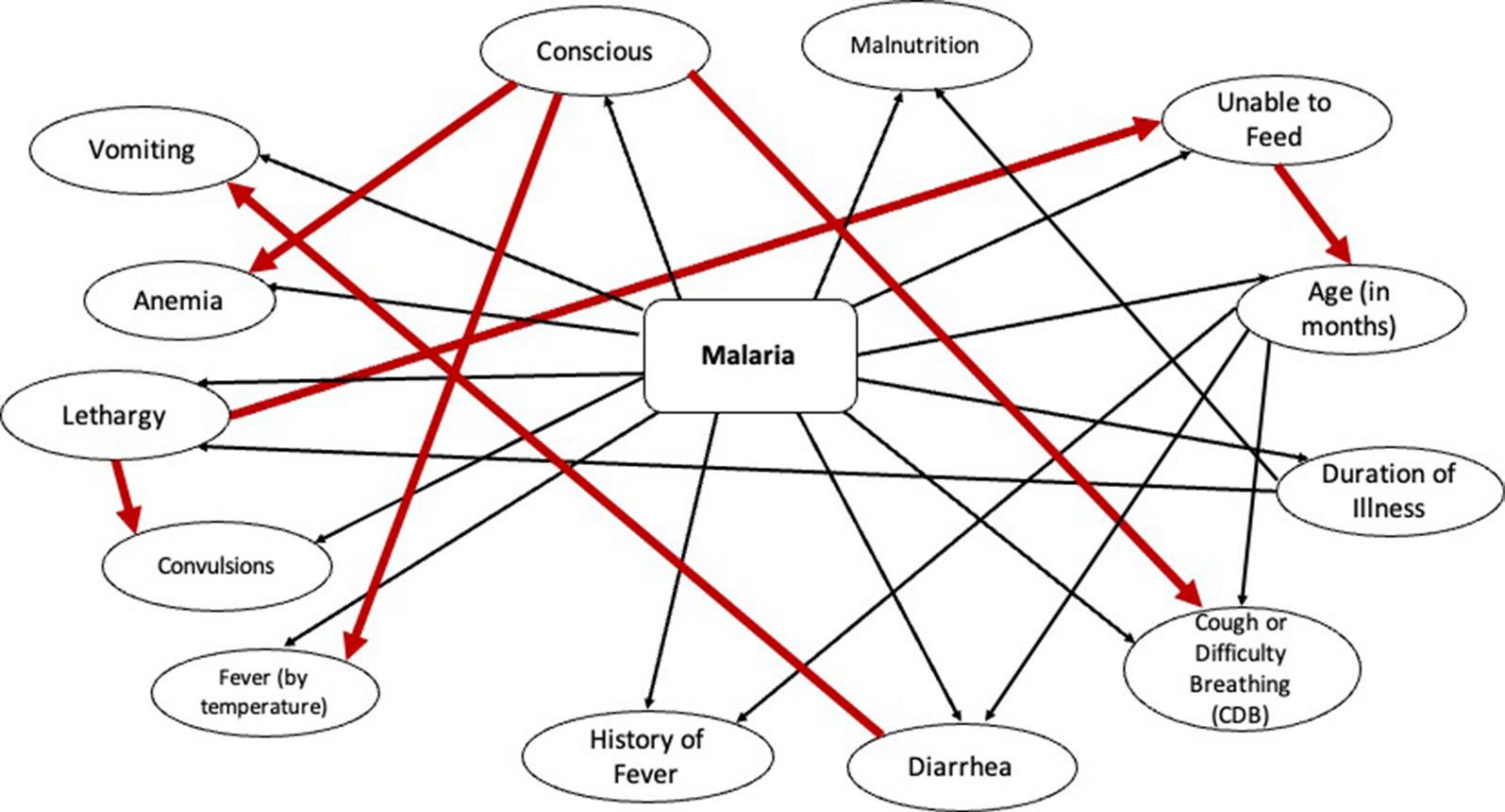
The TAN model derived from the full dataset. The arcs with high strength of influence are colored red

The TAN model is shown in Fig. 3 and was derived from the full dataset. The model contains 14 nodes and 25 arcs. The 7 red arcs in the model indicate associations of high strength of influence. The strength of influence of an arc in the TAN model measures the Euclidean distance between the conditional probability distributions of the nodes linked by that arc [36]. These included association of [1] consciousness with anemia, fever (by temperature), and CDB, [2] lethargy with convulsions and inability to feed, [3] inability to feed with age, and [4] diarrhea with vomiting.

### Performance of models

Table 3 provides a summary of the performance values of all models obtained from tenfold cross-validation at the default threshold of 0.5 and the optimal threshold obtained from maximizing Youden’s Index. See Additional File 1 for additional details, including the confusion matrices, receiver operating characteristic (ROC) curves, and *p* values from statistical tests.

**Table 3:** Performance of the models computed using tenfold cross-validation

Performance metric	Threshold	Manual BN	TAN	Logistic regression	Random forest
Area under ROC curve (AUC)	–	0.60	0.58	0.57	0.57
Balanced accuracy (BAC)	Default	0.56	0.53	0.50	0.50
Sensitivity	Default	0.32	0.22	0.07	0.03
Specificity	Default	0.80	0.84	0.93	0.97
Balanced accuracy (BAC)	Optimal	0.58	0.57	0.57	0.57
Sensitivity	Optimal	0.74	0.45	0.59	0.52
Specificity	Optimal	0.42	0.68	0.55	0.62

Our experiments yielded AUCs of 0.57 (logistic regression, random forest) to 0.60 (manual BN). The manual BN model had statistically significantly better AUC compared to the other models (*p* < 0.05, *p* < 0.05, *p* < 0.05 using the DeLong test for all comparisons).

The manual BN model had the highest BAC at both thresholds (0.56 at the default threshold and 0.58 at the optimal threshold). The manual BN model’s BAC at the default threshold was statistically significantly better when compared to the other models (*p* < 0.006, *p* < 0.049, *p* < 0.027 using the paired Wilcoxon test for all comparisons). However, at the optimal threshold, the manual BN model’s BAC was not statistically significantly better (*p* = 0.846, *p* = 0.375, *p* = 0.769 using the paired Wilcoxon test for all comparisons).

The manual BN model had the highest sensitivity at both thresholds (0.32 at the default threshold and 0.74 at the optimal threshold) and these values were statistically significantly better compared to the other models (*p* < 0.001, *p* < 0.001, *p* < 0.001 using the McNemar’s Chi-Square test at both thresholds for all comparisons). The random forest model had the highest specificity at the default threshold (0.97) that was statistically significantly better than the specificities of the other models (*p* < 0.001, *p* < 0.001, *p* < 0.001 using the McNemar’s Chi-Square test for all comparisons) while the TAN model had the highest specificity at the optimal threshold (0.68) that was statistically significantly better than the specificities of the other models (*p* < 0.001, *p* < 0.001, *p* < 0.001 using the McNemar’s Chi-Square test for all comparisons).

We computed the NRI of TAN, logistic regression, and random forest models compared to the manual BN model. Based on commonly used benchmarks for NRI [31], there is no improvement in any of the models compared to the manual BN model (NRI < 0.2).

### Sensitivity analysis

**Fig. 4:**
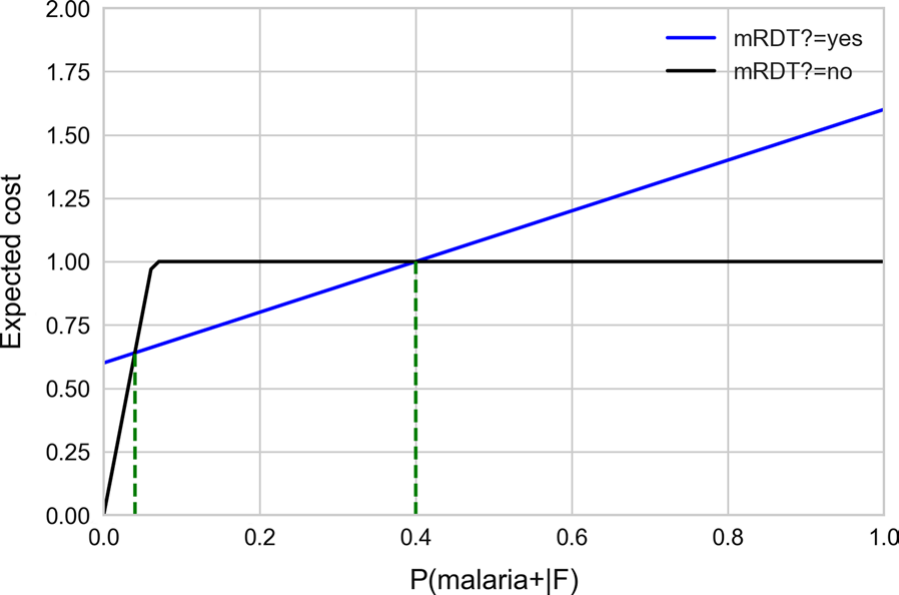
Sensitivity analysis for the decision tree shown in Fig. 1. The probability of malaria is plotted on the x-axis and the expected costs on the y-axis

Figure 4 presents the sensitivity analysis of the decision tree that is shown in Fig. 1. We varied the probability of malaria given the patient findings (x-axis) and computed the expected cost that is plotted on the y-axis. The black and blue lines represent the expected cost of not obtaining and obtaining mRDT (the two branches labeled “no” and “yes” that originate from ‘mRDT?’ in the decision tree), respectively. For the decision of not obtaining mRDT, as the probability of having malaria increased, the expected cost increased and then became constant at US $1.00 at probability 0.0625 and above. And, for the decision of obtaining mRDT, as the probability of having malaria increased, the expected cost increased, and at probability 0.40, this cost surpassed the cost when not obtaining the test. Based on this analysis, when the probability of having malaria is between 0.0 and 0.04 or between 0.40 and 1.00 (Fig. 4), the preferred decision is to forego the test. This is an illustrative analysis to demonstrate judicious use of mRDT in a resource-constrained setting where the availability of mRDT is limited. See Additional File 1 for examples of computing the expected costs.

## Discussion

The current practice for the management of malaria in children involves the use of mRDT and a course of ACT for a child presenting with fever. With the limited availability of both mRDTs and ACT drugs in rural health centers in Malawi and other LMICs, a more sustainable strategy for judicious use of these resources is needed. We developed predictive models that computed the probability of malaria based on clinical findings and developed a simple decision tree to determine the optimal use of mRDT based on the probability of malaria.

To the best of our knowledge, this is the first study to develop BN models for the prediction of childhood malaria in Malawi, which serves as an example of an LMIC. We derived two BN models, including a manually specified model and an automatically derived TAN model. We compared the performance of the BN models to that of logistic regression and random forest models. The manual BN model achieved the highest AUC, BAC, and sensitivity values at both the default and optimal thresholds. The logistic regression and random forest models exhibited low sensitivity values at the default threshold; however, the values improved when using the optimal threshold. The random forest model had the highest specificity at the default threshold, while the TAN model achieved the highest specificity at the optimal threshold. Between the BN models, the TAN model using the optimal threshold with higher specificity might be preferred for the classification of malaria in a resource-constrained setting to enable judicious use of the mRDT for diagnosis. However, the manual BN model using the optimal threshold with higher sensitivity could avoid the high costs resulting from untreated disease.

The BN models provide several advantages over the current malaria management approach. Fever alone has been found to be a poor indicator of childhood malaria [37], and CDB, anemia, malnourishment, and diarrhea have been found to be associated with an increased likelihood of malaria [38, 39]. Since the BN models capture associations in addition to the main ones, such as between fever and malaria, they are more accurate than estimates that are based on a single feature [40]. The manual BN model is simple and interpretable with good performance on several metrics compared to TAN and the other models. Simpler models are easier to interpret and may be preferred for clinical use if their performance is similar or vary only slightly from that of more complex models [16]. Further, during application, BN models can compute predictions even if some of the values of the predictors are missing, though in our study we chose to model missing values explicitly as a special value. In clinical data, missingness can be informative, and modeling the missingness explicitly has been shown to improve performance in BN classifiers [41].

Integration of the probabilities obtained from a predictive model, such as a BN model, with the costs of resources such as tests and drugs in a decision tree can provide the basis of optimal decision making at rural health posts. In the example decision tree that we used (see Fig. 1), we included illustrative costs of mRDT tests and ACT drugs. For values of probability of malaria below 0.04 and above 0.40, sensitivity analysis indicates that the mRDT test can be omitted to minimize expected costs. Thus, accurate estimation of the probability of malaria based on clinical features can lead to judicious use of mRDT, conserving the test for children whose probability of malaria based on clinical features is intermediate (between 0.04 and 0.40). This implies that at probabilities below 0.04 the decision to not treat and at probabilities above 0.40 the decision to treat can be made with high confidence without an mRDT, and the mRDT is most useful at probabilities in the range 0.04 to 0.40. The decision tree in this paper includes illustrative costs of resources, and it is designed to optimize the judicious use of those resources from the viewpoint of the payer of healthcare; however, it is possible to model costs that include other considerations and perspectives.

As Malawi has an emerging Electronic Medical Record System [42, 43], one possibility is to integrate the BN model to provide the probability of malaria to healthcare workers such as HSAs at the point of care to enable them to use mRDT more judiciously.

### Limitations

There are several limitations to our study. The dataset that we used was derived from the SPA survey, and our choice of variables was constrained by the information collected in the survey. For example, the survey did not include details, such as the immunization and HIV status of the children, which are important for determining the risk of malaria. Additionally, the proportion of children with malnutrition in the data was much less than the reported prevalence in the country, which suggests that this variable might have been underreported [44]. Information about prior exposure to anti-malarial drugs would also be useful but was not collected in the survey. Thus, there may exist latent associations among variables that were not captured in our models.

We believe that the choice of using the mRDT result as the gold standard diagnosis is a reasonable approach given the dataset and the WHO reported high sensitivity and specificity of the test. As the type of mRDT and procedure of the test was not made available with the dataset, we cannot verify the reported outcome. We removed the encounters that did not include an mRDT result, which reduced the number of encounters substantially. A smaller dataset limits the reliability of the parameter estimates in all models, including the BN models. Further, the selected dataset may yield biased predictions that are not representative of the outcomes in the remainder of the dataset. However, this is the only dataset that we know of with both gold standard diagnosis and clinical features of childhood malaria available. While this study developed and validated the models with the same dataset (using a cross-validation design), external validation with appropriate feedback from the healthcare providers in Malawi would be valuable to guide the next steps to refine the model for clinical use.

The decision analysis considered only the costs of tests and ACT drugs. The analysis also assumes that if the disease becomes severe, then treatment is provided, albeit, at a higher cost. Additional costs and preferences based on the local needs can be incorporated in the decision tree for more sophisticated decision analysis to make it more applicable for clinical use. The analysis can be also be extended to include several outcomes in the case of progression of the disease to severe complicated malaria and death [8].

## Conclusions

Current clinical guidelines for the management of childhood malaria in LMICs such as Malawi are based on WHO guidelines that require that a child receive a confirmatory diagnosis based on microscopy or mRDT before deciding to put the child on a course of ACT. However, in resource-constrained settings, mRDT and ACT drugs may not always be available. Thus, a clinical decision support system that provides personalized guidance on when to use mRDT could aid the healthcare worker in conserving the use of mRDT.

We used clinical features from a publicly available dataset to derive models that predict malaria in an LMIC setting. Integration of predictions with costs of resources, such as mRDTs and ACT drugs, in a decision tree provides a way to model the rationale use of those resources. The application of such models at the point of care will require the development of clinical decision support that can provide nuanced guidance for the personalized management of childhood malaria.

## Supplementary Information

**Additional file 1**. Additional results for the performance of the models including confusion matrices, ROC curves, results of statistical tests as well as examples of computation of expected costs for the decision tree.

## Data Availability

The SPA dataset used for analysis in this study is publicly available through the DHS website, https://www.dhsprogram.com/. A subset of the data extracted by the authors is available from the corresponding author on reasonable request.

## Abbreviations

ACT: Artemisinin Combination Therapy
AUC: Area under the Receiving Operating Characteristic curve
BN: Bayesian network
CDB: Cough or difficulty breathing
DHS: Demographic and Health Surveys
eCDA: Electronic clinical decision algorithm
HSA: Health surveillance assistant
LMIC: Low- and middle-income country
mRDT: Malaria rapid diagnostic test
NGO: Non-governmental organization
NRI: Net reclassification improvement
SPA: Service Provision Assessment
TAN: Tree Augmented Naïve Bayes
WHO: World Health Organization
