# Supplementary material for: Bayesian network models with decision tree analysis for management of childhood malaria in Malawi
Source: BMC Med Inform Decis Mak. 2021 May 17;21:158. doi: 10.1186/s12911-021-01514-w (PMC8130361; doi:10.1186/s12911-021-01514-w)
Supplement: Supplementary file 1 — Additional file 1. Additional results for the performance of the models including confusion matrices, ROC curves, results of statistical tests as well as examples of computation of expected costs for the decision tree. [file 12911_2021_1514_MOESM1_ESM.docx]

**Additional file 1**

We provide additional results for the performance of the models including confusion matrices, ROC curves and the results of statistical tests. In addition, we provide examples of computation of expected costs for the decision tree.

**A.1 Confusion Matrices**

**Table A1:** Confusion matrix for the manual BN model (with default threshold).

|  | **Predicted malaria absent** | **Predicted malaria present** |
| --- | --- | --- |
| **Actual malaria absent** | 583 | 141 |
| **Actual malaria present** | 282 | 133 |

**Table A2:** Confusion matrix for the manual BN model (with optimal threshold).

|  | **Predicted malaria absent** | **Predicted malaria present** |
| --- | --- | --- |
| **Actual malaria absent** | 301 | 423 |
| **Actual malaria present** | 109 | 306 |

**Table A3:** Confusion matrix for the TAN model (with default threshold)

|  | **Predicted malaria absent** | **Predicted malaria present** |
| --- | --- | --- |
| **Actual malaria absent** | 607 | 117 |
| **Actual malaria present** | 322 | 93 |

**Table A4:** Confusion matrix for the TAN model (with optimal threshold)

|  | **Predicted malaria absent** | **Predicted malaria present** |
| --- | --- | --- |
| **Actual malaria absent** | 492 | 232 |
| **Actual malaria present** | 226 | 189 |

**Table A5:** Confusion matrix for the logistic regression model (with default threshold)

|  | **Predicted malaria absent** | **Predicted malaria present** |
| --- | --- | --- |
| **Actual malaria absent** | 674 | 50 |
| **Actual malaria present** | 387 | 28 |

**Table A6:** Confusion matrix for the logistic regression model (with optimal threshold)

|  | **Predicted malaria absent** | **Predicted malaria present** |
| --- | --- | --- |
| **Actual malaria absent** | 397 | 327 |
| **Actual malaria present** | 171 | 244 |

**Table A7:** Confusion matrix for the random forest model (with default threshold)

|  | **Predicted malaria absent** | **Predicted malaria present** |
| --- | --- | --- |
| **Actual malaria absent** | 703 | 21 |
| **Actual malaria present** | 402 | 13 |

**Table A8:** Confusion matrix for the random forest model (with optimal threshold)

|  | **Predicted malaria absent** | **Predicted malaria present** |
| --- | --- | --- |
| **Actual malaria absent** | 447 | 277 |
| **Actual malaria present** | 200 | 215 |

**A.2 ROC Curves**


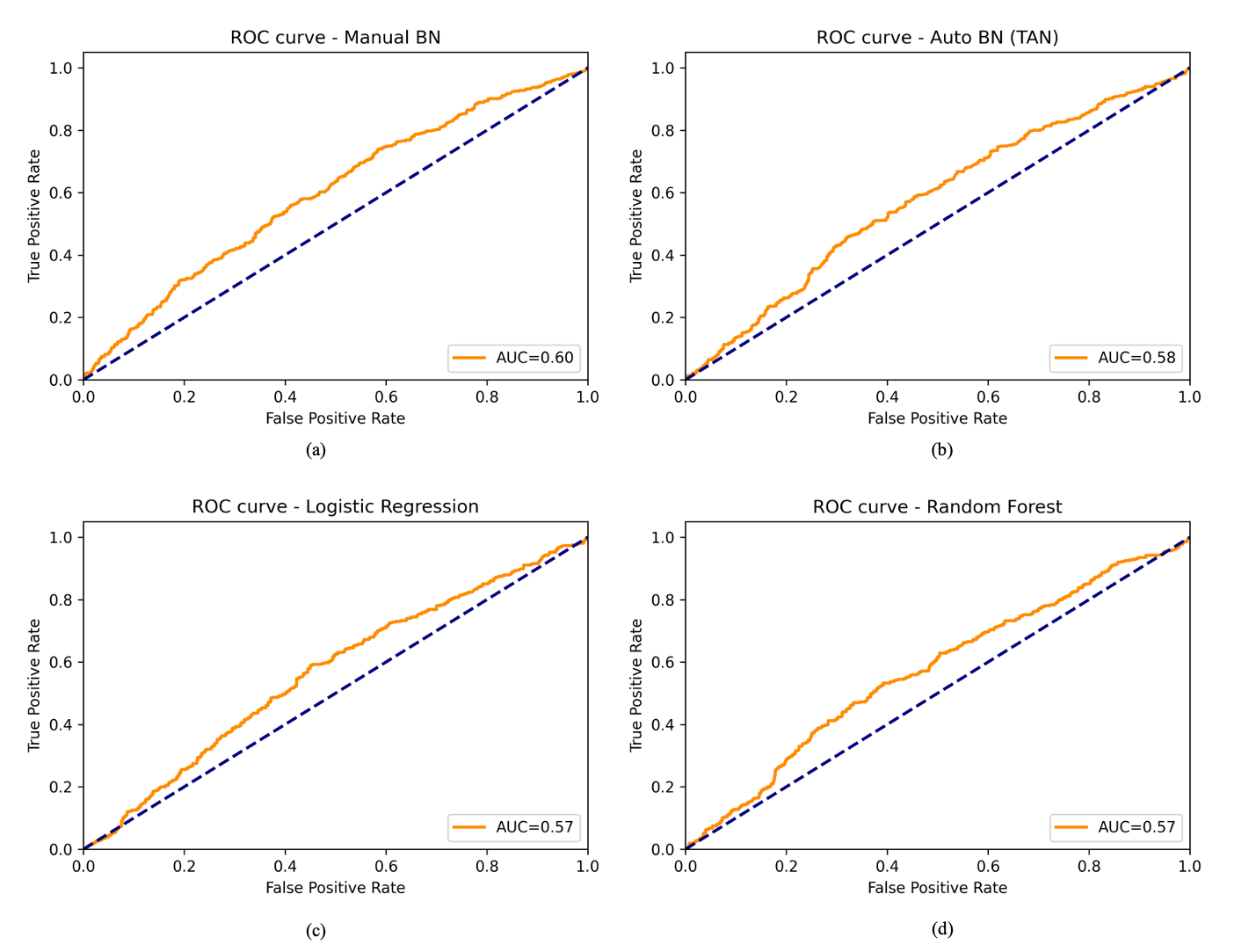


**Figure A1:** ROC curves for the manual BN, TAN, logistic regression, and random forest models.

**A.3 Results of Statistical Tests**

**Table A9:** p-values from pairwise comparisons of AUCs of the models using the DeLong test.

| **AUC** | **Experimental model** | | |
| --- | --- | --- | --- |
| **Baseline model** | Random Forest | Manual BN | TAN |
| Logistic Regression | 0.492 | **0.004** | 0.289 |
| Random Forest | - | **0.003** | 0.471 |
| Manual BN | - | - | **0.042** |

**Table A10:** p-values from pairwise comparisons of BACs of the models using the paired two-sample Wilcoxon test (with default threshold).

| **BAC (with default threshold)** | **Experimental model** | | |
| --- | --- | --- | --- |
| **Baseline model** | Random Forest | Manual BN | TAN |
| Logistic Regression | 1.000 | **0.006** | **0.037** |
| Random Forest | - | **0.049** | 0.126 |
| Manual BN | - | - | **0.027** |

**Table A11:** p-values from pairwise comparisons of BACs of the models using the paired two-sample Wilcoxon test (with optimal threshold).

| **BAC (with optimal threshold)** | **Experimental model** | | |
| --- | --- | --- | --- |
| **Baseline model** | Random Forest | Manual BN | TAN |
| Logistic Regression | 0.922 | 0.846 | 1.000 |
| Random Forest | - | 0.375 | 0.922 |
| Manual BN | - | - | 0.769 |

**Table A12:** p-values of pairwise comparisons of the sensitivity values of the models using the McNemar's Chi-Square test (with default threshold).

| **Sensitivity (with default threshold)** | **Experimental model** | | |
| --- | --- | --- | --- |
| **Baseline model** | Random Forest | Manual BN | TAN |
| Logistic Regression | **0.003** | **<0.001** | **<0.001** |
| Random Forest | - | **<0.001** | **<0.001** |
| Manual BN | - | - | **<0.001** |

**Table A13:** p-values of pairwise comparisons of the sensitivity values of the models using the McNemar's Chi-Square test (with optimal threshold).

| **Sensitivity (with optimal threshold)** | **Experimental model** | | |
| --- | --- | --- | --- |
| **Baseline model** | Random Forest | Manual BN | TAN |
| Logistic Regression | **<0.001** | **<0.001** | **<0.001** |
| Random Forest | - | **<0.001** | **<0.001** |
| Manual BN | - | - | **<0.001** |

**Table A14:** p-values of pairwise comparisons of the specificity values of the models using the McNemar's Chi-Square test (with default threshold).

| **Specificity (with default threshold)** | **Experimental model** | | |
| --- | --- | --- | --- |
| **Baseline model** | Random Forest | Manual BN | TAN |
| Logistic Regression | **<0.001** | **<0.001** | **<0.001** |
| Random Forest | - | **<0.001** | **<0.001** |
| Manual BN | - | - | 0.011 |

**Table A15:** p-values of pairwise comparisons of the specificity values of the models using the McNemar's Chi-Square test (with optimal threshold).

| **Specificity (with optimal threshold)** | **Experimental model** | | |
| --- | --- | --- | --- |
| **Baseline model** | Random Forest | Manual BN | TAN |
| Logistic Regression | **<0.001** | **<0.001** | **<0.001** |
| Random Forest | - | **<0.001** | **<0.001** |
| Manual BN | - | - | **<0.001** |

**Table A16:** Net reclassification improvement (NRI) with the default threshold.

| **NRI (with default threshold)** | **Experimental model** | | |
| --- | --- | --- | --- |
| **Baseline model** | Random Forest | Manual BN | TAN |
| Logistic Regression | 0.004 | 0.127 | 0.064 |
| Random Forest | - | 0.123 | 0.060 |
| Manual BN | - | - | -0.063 |

**Table A17:** Net reclassification improvement (NRI) with the optimal threshold.

| **NRI (with optimal threshold)** | **Experimental model** | | |
| --- | --- | --- | --- |
| **Baseline model** | Random Forest | Manual BN | TAN |
| Logistic Regression | -0.001 | 0.017 | -0.001 |
| Random Forest | - | 0.018 | -0.001 |
| Manual BN | - | - | -0.018 |

**A.4 Examples of Computation of Expected Costs for the Decision Tree**

As illustrative examples, we computed the expected costs for three encounters in the dataset for the decision tree shown in Figure 1. For each of the encounters, we obtained the probability of having malaria from the manual BN model. Table A18 shows the values for the predictor variables and the value of the target variable from the manual BN model.

**Table A18:** Predictor variable values and probability of malaria for three encounters.

| **Predictor variable** | **Encounter 1** | **Encounter 2** | **Encounter 3** |
| --- | --- | --- | --- |
| Age (in months) | Other | 2-12 | 24-60 |
| Duration of Illness (in days) | 15-30 | 3-15 | 3-15 |
| Conscious | Yes | Yes | Yes |
| Anemia | Absent | Absent | Present |
| Convulsions | Absent | Absent | Absent |
| Cough or Difficulty Breathing (CDB) | Present | Present | Absent |
| Diarrhea | Absent | Present | Present |
| History of Fever | Absent | Present | Present |
| Fever (temperature>37.5 C) | Absent | Absent | Present |
| Lethargy | Absent | Absent | Absent |
| Malnutrition | Absent | Absent | Absent |
| Unable to Feed | Absent | Absent | Present |
| Vomiting | Absent | Absent | Present |
| **Target variable** |  |  |  |
| P(malaria+\|F) | 0.018 | 0.202 | 0.745 |

Given the probability of malaria for an encounter, we computed the expected costs of performing and not performing the mRDT that are represented by the [mRDT?=no] and [mRDT?=yes] branches of the decision tree in Figure 1. We use the equations described in Section 2.6 on Decision Tree Development to compute the costs. Table A19 displays the expected costs of the [mRDT?=yes] and [mRDT?=no] branches in the decision tree for each encounter. For encounters 1 and 3, the expected cost of the [mRDT?=no] branch is lower than the [mRDT?=yes] branch and the preferred decision is to forego the mRDT. These encounters serve as examples where P(malaria+|F) lies between 0 and 0.04, and 0.40 and 1.00 respectively (see Figure 4). However, for encounter 2, the expected cost of obtaining the mRDT is lower than the expected cost of not obtaining the mRDT and thus, the preferred decision is to perform the test. This encounter serves as an example where P(malaria+|F) lies between 0.04 and 0.40 (see Figure 4).

**Table A19:** Probability of malaria and expected costs of performing and not performing the mRDT for three encounters.

|  | **P(malaria+\|F)** | **Expected cost of [mRDT?=no]** | **Expected cost of [mRDT?=yes]** |
| --- | --- | --- | --- |
| **Encounter 1** | 0.018 | **0.288** | 0.618 |
| **Encounter 2** | 0.202 | 1.000 | **0.802** |
| **Encounter 3** | 0.745 | **1.000** | 1.345 |
